# The association of BMI, lifestyle behaviors, and knowledge of acute cholecystitis of adults: a cross-sectional study

**DOI:** 10.7717/peerj.21172

**Published:** 2026-05-04

**Authors:** Maha Ali, Amani Alhazmi, Ali Mohieldin, Waled A. M. Ahmed, Mahmoud Abdelwahab Khedr, Osman Babiker Osman Mohammed, Manal Mohammed Hawash, Faroq Abdulghani Alshameri, Nahid Khalil Elfaki, Ghala Abdullah Alharbi

**Affiliations:** 1 Department of Public Health, College of Applied Medical Sciences, King Khalid University, Abha, Saudi Arabia; 2 Community Health Nursing Department, Faculty of Nursing, Al-Baha University, Al-Baha, Saudi Arabia; 3 Psychiatric and Mental Health Nursing Department, Alexandria University, Alexandria, Egypt; 4 Department of Public Health, Faculty of Applied Medical Sciences, Al-Baha University, Al-Baha, Saudi Arabia; 5 Medical and Surgical Nursing Department, Faculty of Nursing, University of Tabuk, Tabuk, Saudi Arabia; 6 Community Health Nursing Department, College of Nursing, Najran University, Najran, Saudi Arabia; 7 College of Medicine, King Khalid University, Abha, Saudi Arabia

**Keywords:** Acute cholecystitis, BMI, Lifestyle behaviours, Health knowledge, Adults

## Abstract

**Objective:**

Acute cholecystitis is a common biliary disorder with rising prevalence in Saudi Arabia, yet the relationship between body mass index (BMI), lifestyle behaviors, and public knowledge about the condition remains underexplored. This study assessed these associations to inform preventive and educational strategies.

**Methods:**

A cross-sectional survey was conducted among 385 adults aged 18–80 years residing in Abha, Saudi Arabia, who voluntarily agreed to participate. Data were collected using a validated, self-administered online questionnaire covering socio-demographics, lifestyle behaviors, and four knowledge domains. Knowledge levels were categorized using a validated scoring system in which correct responses were summed and classified as low, moderate, or high based on predetermined cutoff scores. BMI was calculated from self-reported height and weight.

**Results:**

Participants were predominantly female (76.1%), The mean age of participants was 33 ± 14 years, and their mean BMI was 27.5 ± 5 kg/m^2^, indicating a generally young sample with a high prevalence of overweight and obesity. Knowledge was moderate in most respondents (53.2%), high in 43.9%, and low in 2.9%. Risk factor and symptom knowledge scored highest (mean = 2.40/3), while dietary risk knowledge scored lowest (mean = 2.13/3). BMI correlated positively with lifestyle score (r = 0.310, *p* = 0.041), dietary risk knowledge (r = 0.280, *p* = 0.047), and risk factor knowledge (r = 0.360, *p* = 0.008). Lifestyle scores correlated significantly with all knowledge domains (r = 0.390–0.450, *p* < 0.01). Although the correlation coefficients were modest (r ≤ 0.40), they still indicate meaningful but limited shared variance, suggesting that BMI and lifestyle factors contribute only partially to differences in knowledge. Most participants favoured periodic check-ups (26.2%) and laparoscopic surgery (25.5%) as preferred treatments.

**Conclusion:**

Adults in Abha generally demonstrate moderate awareness of acute cholecystitis, with notable gaps in dietary risk and prevention knowledge. Higher BMI and healthier lifestyle behaviors are associated with greater knowledge in certain domains. Future studies should employ representative sampling methods and multivariable analytical approaches to better examine independent relationships between lifestyle factors and disease awareness.

## Background

Acute cholecystitis is an inflammatory condition of the gallbladder primarily caused by cystic duct obstruction, often resulting from gallstones (Gallaher & Charles, 2022). If left untreated, it can lead to complications such as gallbladder perforation or gangrene. It is estimated to affect 10% to 20% of adults worldwide, with a rising prevalence in Saudi Arabia (Badoghaish et al., 2023). Especially among females and people with obesity, physical inactivity and dietary risk factors. However, presentation symptoms (such as right upper quadrant abdominal pain, loss of appetite, fever, indigestion, nausea and vomiting) can be similar to those seen in other abdominal and thoracic diseases (such as peptic ulcers, irritable bowel syndrome, cardiac ischemia or acute/chronic pancreatitis), potentially leading to delays in diagnosis (Alessa et al., 2025; Wang et al., 2024). Although laparoscopic cholecystectomy is the recommended option, conservative management—comprising pain relief, intravenous fluids, and antibiotics—could be considered for patients who are high-risk surgical candidates or those with mild, uncomplicated symptoms (Al Atsariyah et al., 2022; Chauhan et al., 2019; Mirghani et al., 2022; Stokes & Lammert, 2021; Wang et al., 2024).

Due to the increase in the prevalence of cholecystitis in Saudi Arabia, cholecystectomy is one of the most frequently performed major abdominal surgeries in the country (Alanazi et al., 2018; Mahfouz et al., 2022). Recent epidemiologic studies have demonstrated a gradual increase in the incidence of gallstone disease and acute cholecystitis globally, notably affecting high- and middle-income countries with a documented increase in incidence since 2005 (Wang et al., 2024). These trends have also been seen in Saudi Arabian society and are related to the increasing levels of obesity and shifts toward high-calorie diets and sedentary behavior. Acute cholecystitis is more prevalent among females than males, with an approximate female to male ratio of 2–3:1, showing a marked increase after the fifth decade of age (Alessa et al., 2025). Risk factors for gallstone disease include being female, middle-aged (in the 40 s), overweight, and having conditions like diabetes or hyperlipidaemia, which are commonly summarized as the “four Fs”: Female, Forties, Fat, and Fertile (Alanazi et al., 2018; Zhang et al., 2023).

The underlying cause of acute cholecystitis is inflammation resulting from cystic duct obstruction, which leads to alterations in bile concentration (Gallaher & Charles, 2022). Normally, bile produced by the liver is transported *via* the bile duct and stored in the gallbladder, where it is concentrated (Jones et al., 2023). When bile concentration is disrupted due to bile stasis, the supersaturation of lipids and cholesterol in the liver, or other disturbances, gallstones can form. Biliary colic may progress to acute calculous cholecystitis if obstruction persists. In cases where gallstones are absent despite clinical inflammation, the condition is termed acute acalculous cholecystitis (Kumar et al., 2025; Rodríguez-Antonio et al., 2020).

While gallstones are found in approximately 95% of patients with acute cholecystitis, not all cases require treatment; only about 20% of individuals with asymptomatic gallstones will develop symptoms over 20 years, and only 1% of these may face complications before symptoms appear (Gallaher & Charles, 2022). The diagnosis of cholecystitis involves a detailed physical examination and a thorough medical history. Tests such as a complete blood count are important; a high white blood cell (WBC) count can indicate acute cholecystitis or a more severe complications such as gangrenous cholecystitis or gallbladder perforation, while elevated liver enzyme levels may suggest a common bile duct stone, especially if bilirubin levels exceed two mg/dL (Doherty et al., 2022; Gallaher & Charles, 2022; Mencarini et al., 2024).

The diagnosis of acute cholecystitis may be difficult, as normal laboratory values do not exclude advanced gallbladder disease (Evanson et al., 2025; Jean-Antony, 2022). Ultrasound is often used in the initial evaluation because of its high sensitivity for gallstones and a thickened gallbladder wall, whereas CT tends to be used more frequently in the emergency department setting for broader assessment (Malik et al., 2021; Radmard et al., 2015).

The standard treatment for cholecystitis is laparoscopic cholecystectomy, which is favored for its low morbidity and mortality rates and quicker recovery times (Mannam et al., 2023; Mencarini et al., 2024). For critically ill patients deemed unfit for surgery, percutaneous drainage of the gallbladder serves as an alternative temporary measure to alleviate symptoms (Jean-Antony, 2022; Popowicz, 2023). In cases of less severe chronic cholecystitis, particularly for patients who are not candidates for surgery, a low-fat, low-spice diet may be recommended, although outcomes can vary (Kohga et al., 2019; Palamuthusingam et al., 2025; Yun & Seo, 2018).

Previous research, including that by Al Atsariyah et al. (2022), suggests that the prevalence of cholecystitis varies within Saudi Arabia but the association between body mass index (BMI) and types of cholecystitis was not investigated. Research by Zhang et al. (2023), with 125,668 Chinese adults emphasizes that the risk of developing gallstones is associated with both modifiable factors (obesity, diet, medication use, and physical inactivity) and non-modifiable factors (age, ethnicity, genetic predisposition and sex) (Zhang et al., 2023). Strong and significant associations have been found between high BMI and the symptomatic form of gallstone diseases in various populations (Kumar et al., 2025; Malik et al., 2021; Rodríguez-Antonio et al., 2020), and obesity has been identified as a major risk factor for cholesterol gallstones because it increases hepatic cholesterol excretion (Radmard et al., 2015).

Previous literature (Parra-Landazury, Cordova-Gallardo & Méndez-Sánchez, 2021) has investigated BMI, lifestyle and physical activity patterns and how these may be related to the knowledge and behaviors of Saudi citizens, especially at university level. These results reveal a relationship between modifiable lifestyle behaviors and gallstone disease, but little data exist on how BMI, lifestyle behaviors and disease knowledge are associated with acute cholecystitis (Malik et al., 2021; Rindler, Gries & Freidl, 2023; Zhang et al., 2023). The combined evaluation of these factors (as in this current study) is original and yields new knowledge on how modifiable lifestyle behaviors interact with BMI to impact the prevalence and experience of gallstone symptoms. This approach addresses a recognized gap in the regional literature and provides useful data for preventive medicine and health promotion (Almetrek et al., 2022). Physical activity has been recognized as a significant modifiable risk for gallstone disease and its complications, including acute cholecystitis. Regular physical activity has been significantly associated to enhanced lipid turnover, increased insulin sensitivity and lower biliary cholesterol saturation, thereby reducing the risk of developing gallstones (Qian et al., 2022; Sun et al., 2022). This study aims to assess Saudi adults’ knowledge of acute cholecystitis, including its symptoms and risk factors, and investigate the relationship between BMI, lifestyle behaviours (diet, physical activity, smoking), and knowledge of the disease. Accordingly, the research seeks to answer the following questions: What is the level of Saudi adults’ knowledge about acute cholecystitis, and how does it vary by demographics? How do BMI and lifestyle behaviours relate to this knowledge?

## Methods

**Study design:** This cross-sectional study was conducted among the general population of Abha, the capital of the Asir Region in south-western Saudi Arabia. Abha is located at an altitude of about 2,270 m above sea level and is recognized for its moderate climate and rich cultural heritage, making it a key urban center in the region (Awan et al., 2021; Saudi Tourism Authority, 2024; Kingdom of Saudi Arabia-Vision 2030, 2025).

**Study population and inclusion criteria:** The study included residents of Abha, aged between 18 and 80 years, who were able to read Arabic and consented to participate in the study. Those who did not agree or had intellectual disabilities, and those unable to complete an Arabic-language online survey were excluded from participation. The questionnaire was administered exclusively in Arabic, following professional translation and cultural adaptation.

**Study period and setting:** Data were collected over a 3-month period from April to June 2025 in Saudi Arabia.

**Sampling and sample size:** The study sample was obtained using convenience sampling through social media and digital networks. The sample size was calculated using the Raosoft sample size calculator, with a 95% confidence level and a 5% margin of error (Raosoft, 2020). The sample size was calculated according to the following primary outcome: to assess adult knowledge about acute cholecystitis and related lifestyle behaviors among adults in Abha. The 2022 census data indicate that the total adult population of Abha numbered 334,290 (General Authority for Statistics, 2023). According to these characteristics, a minimum sample size of 384 respondents was estimated. The eventual sample size (*n* = 385) was slightly higher than the calculated one, ensuring a fair degree of accuracy for estimations and power for the intended inferential analyses.

### The validity and reliability of the survey instrument

A pilot study was conducted with 40 participants from the target population to evaluate the validity and reliability of the questionnaire, which was completed anonymously and supervised to ensure valid responses. No further changes were needed, as the final questionnaire had already been revised based on student feedback. Cronbach’s alpha was equal to 0.706, which suggests acceptable reliability. The constructs “Knowledge about risk factors” and “Knowledge about symptoms” demonstrated high internal consistency, with Cronbach’s alpha values of 0.802 and 0.805, respectively, each based on seven items. The construct “Knowledge about the prevention of acute cholecystitis” showed a reliability score of 0.733 for six items. The pilot study results indicated that the scales were clear and effective at capturing the intended constructs. Overall, the results suggested that all constructs had acceptable to good reliability, supporting the internal consistency of the survey instrument. The participants involved in the pilot study were excluded from the main study’s sample.

### Instruments of data collection

Data for this study were gathered *via* a structured, self-administered questionnaire, originally developed by Alghamdi et al. (2018) and meticulously translated into Arabic by a qualified translator to ensure cultural and linguistic appropriateness. It was administered online through Google Forms from April to June 2025.

The survey link was distributed through community WhatsApp groups, university networks, and widely used social media platforms in Abha (Twitter/X and Instagram). Residents who met the inclusion criteria and wished to participate voluntarily could access and complete the survey anonymously. The survey was available for a clearly defined 3-month period which served as the official data-collection timeframe. This method enabled the research team to reach a wide community segment while maintaining flexibility, confidentiality, and anonymity for participants.

The questionnaire was structured into three sections: the first section collected socio-demographic information, including participants’ city of residence, age group, gender, marital status, education level, and occupation. The second explored lifestyle behaviors, such as nutrition, physical activity, smoking, weight management, sleep patterns, stress levels, and caffeine consumption. It also included self-reported height and weight to calculate each participant’s BMI using the World Health Organization (WHO) method, aiming to identify personal and behavioural factors potentially associated with acute cholecystitis (Davies et al., 2020).

The third section assessed knowledge of acute cholecystitis in five domains: dietary factors, risk determinants, common symptoms, preventive practices and treatment options. Knowledge was captured as multiple-choice and true/false items. One point was given for each correct answer, resulting in an objective knowledge score between 0 and 20. Moreover, we used an additional 25-item Likert-type scale to capture perceived understanding and awareness concerning the condition. By design this dual scoring system served both a descriptive and perception-based purpose but should be interpreted in context as the thresholds for encoding into knowledge categories were operational and descriptive. While internal consistency was assessed, the tool does not measure clinical knowledge *vs* general health literacy or exposure to health-related questionnaires. Thus, knowledge score should be considered an indication of general awareness and relative among the study samples instead of validated clinical knowledge.

### Statistical methods

The socio-demographic profiles, lifestyle behaviors, and knowledge of acute cholecystitis of the 385 participants were analyzed using SPSS v.26. (IBM Corp., Armonk, NY, USA). Categorical and continuous variables were summarized using descriptive statistics through frequencies, percentages, means, and standard deviations. Data distribution was evaluated using the Shapiro–Wilk test to assess normality. The composite scores demonstrated approximate normality, supporting the use of Pearson’s correlation coefficients for inferential analysis. Participants’ awareness of acute cholecystitis was evaluated across four domains—dietary factors, risk determinants, symptoms, and prevention—using a structured 3-point Likert scale. Domain-specific composite scores were calculated and categorized into low, moderate, and high awareness levels. Bivariate Pearson correlations were initially computed to examine associations among BMI, lifestyle behavior scores, and knowledge domains. Given the exploratory nature of the study and the possibility of confounding, the manuscript now clarifies that these correlations indicate associative patterns rather than causal or clinically substantive relationships. Statistical significance was set primarily set at *p* < 0.05. However, *p* < 0.01 was also utilized to highlight correlations with higher statistical significance.

### Ethical requirements

The study was conducted in accordance with the Declaration of Helsinki and received ethical approval from the Research Ethics Committee at King Khalid University, referenced as No. ECM#2024-3163. In the introduction to the questionnaire, participants were informed about the purpose of the study and the procedures involved. They were assured that participation was entirely voluntary, with full guarantees of anonymity and confidentiality, and were informed that all collected data would be used solely for research purposes. They provided written consent before participating in the study. All items in the STROBE Statement checklist were considered.

## Results

### Demographic characteristics

Table 1 shows the socio-demographic profiles of the 385 participants. The largest group was in the 29–39 age range (37.1%), and the second biggest group was the 18–28 age range (36.1%). In contrast, the number of respondents in the older age groups was much lower, with 15.8% of participants aged 40–49 years, and just 7% aged 50–59. No respondents were over 60 years (0.0%). The sample was primarily female (76.1%), and mostly single (46.5%). With respect to education, 53% had higher education, 29.6% had finished secondary education, and 6% did not have any formal education. In terms of employment, students comprised the biggest occupational group (38.7%), followed by employees (30.9%) and the unemployed (20%).

**Table 1:** Socio-demographic characteristics of the participants (*n* = 385).

Variable	Categories	Frequency	Percent
City	Abha	376	97.7
	Other	9	2.3
Age (years)	18–28	139	36.1
	29–39	143	37.1
	40–50	52	13.5
	51–61	25	6.5
	40–50	4	10.0
	>72	12	3.1
Gender	Female	293	76.1
	Male	92	23.9
Marital status	Divorced	28	7.3
	Married	145	37.7
	Unmarried	179	46.5
	Widowed	33	8.6
Education level	No formal education	23	6.0
	Elementary	44	11.4
	Secondary	114	29.6
	Higher education	204	53.0
Occupation	Employee	119	30.9
	Retired	40	10.4
	Student	149	38.7
	Unemployed	77	20.0
Frequency of eating fried or fatty foods	Rarely or never	88	22.9
	A few times a month	137	35.6
	Several times a week	58	15.1
	Daily	102	26.4
Frequency of physical activity (≥150 min/week)	Rarely or never	59	15.3
	Occasionally	190	49.4
	Regularly	136	35.3
History of weight loss attempts for health reasons	Yes	311	80.8
	No	74	19.2
BMI (kg/m^2^)	Underweight (Less than 18.5)	20	5.2
	Normal weight (18.5–24.9)	85	22.1
	Overweight (25–29.9)	115	29.8
	Obese Class I (Moderate) (30–34.9)	95	24.7
	Obese Class II (Severe) (35–39.9)	30	7.8
	Obese Class III (Very severe) (40 and above)	40	10.4

### Anthropometric measures

Table 2 indicates that more than two-thirds (68.1%) reported actively engaging in weight-management efforts, while about one-fifth (21.0%) were not attempting such efforts, and 10.9% were unsure. The majority (81.8%) had experienced rapid weight loss in the past year, compared to less than one-fifth (18.2%) who had not. Regarding meal patterns, more than one quarter (27.0%) frequently skipped meals, nearly half (45.5%) did so sometimes, while smaller proportions reported rarely (16.6%) or never (10.9%) skipping meals. A high intake of refined carbohydrates was very common among nearly one quarter (23.6%) of participants and they were sometimes consumed by more than two-fifths (43.1%), while less than one-fifth reported rare (18.7%) or no intake (14.5%).

**Table 2:** Lifestyle behavior of the participants (*n* = 385).

Variable	Categories	Frequency	Percent
Current weight management efforts	Yes	262	68.1
	Not sure	42	10.9
	No	81	21.0
Rapid weight loss in the past year	Yes	315	81.8
	No	70	18.2
Skipping meals or irregular eating patterns	Yes, frequently	104	27.0
	Sometimes	175	45.5
	Rarely	64	16.6
	Never	42	10.9
High intake of refined carbohydrates	Yes, very often	91	23.6
	Sometimes	166	43.1
	Rarely	72	18.7
	Never	56	14.5
Current smoking status	No	282	73.2
	Yes	103	26.8
Adequate intake of fruits, vegetables, and whole grains	Sometimes	193	50.1
	Rarely	26	6.8
	Never	78	20.3
	Daily	88	22.9
Average nightly sleep duration	<5 h	120	31.2
	5–6 h	25	6.5
	7–8 h	137	35.6
	>8 h	103	26.8
Perceived stress level in the past month	Very low	62	16.1
	Low	108	28.1
	Moderate	21	5.5
	High	71	18.4
	Very high	123	31.9
Frequency of caffeinated beverage consumption	Rarely or never	74	19.2
	Several times a month	73	19.0
	Several times a week	142	36.9
	Daily	96	24.9
Typical number of meals per day	One meal	147	38.2
	Two meals	66	17.1
	Three meals	119	30.9
	>three meals	53	13.8

### Knowledge about cholecystitis

The respondents’ knowledge about acute cholecystitis was measured across four main constructs. Each construct was measured using multiple items, but only the aggregated results are displayed for clarity. These results revealed that overall knowledge was moderate across all domains. In detail, knowledge of risk factors was the highest (mean = 2.40) compared to knowledge of prevention (mean = 2.22) and dietary ingredients (mean = 2.13). Knowledge scores were categorized into three levels: low (25–42), moderate (43–58), and high (59–75). The majority of respondents (53.2%; *n* = 205) demonstrated moderate knowledge, while a substantial proportion (43.9%; *n* = 169) achieved high knowledge scores, reflecting strong awareness of risk factors, symptoms, and related aspects of acute cholecystitis. Low knowledge was present in only 2.9% of the sample.

### Relationships between demographics and knowledge about cholecystitis

Table 3 displays the correlations between BMI, lifestyle behaviors, and the four knowledge domains related to acute cholecystitis. BMI has positive significant correlations with lifestyle score (r = 0.310, *p* = 0.041), knowledge of dietary risk (r = 0.280, *p* = 0.047), and risk factors (r = 0.360, *p* = 0.008). Lifestyle score was strongly and significantly correlated with knowledge domains, including dietary risk (r = 0.450, *p* < 0.001), risk factors (r = 0.420, *p* < 0.001), symptoms (r = 0.390, *p* = 0.002), and prevention (r = 0.440, *p* < 0.001).

**Table 3:** The association between BMI, lifestyle behaviors, and knowledge of acute cholecystitis in adults.

Variables	BMI	Lifestyle score	Knowledge of dietary risk	Knowledge of risk factors	Knowledge of symptoms	Knowledge of prevention
BMI	1	0.310* (*p* = 0.041)	0.280* (*p* = 0.047)	0.360** (*p* = 0.008)	0.240 (*p* = 0.064)	0.200 (*p* = 0.088)
Lifestyle score	0.310* (*p* = 0.041)	1	0.450** (*p* < 0.001)	0.420** (*p* < 0.001)	0.390** (*p* = 0.002)	0.440** (*p* < 0.001)
Knowledge of dietary risk	0.280* (*p* = 0.047)	0.450** (*p* < 0.001)	1	0.570** (*p* < 0.001)	0.510** (*p* < 0.001)	0.500** (*p* < 0.001)
Knowledge of risk factors	0.360** (*p* = 0.008)	0.420** (*p* < 0.001)	0.570** (*p* < 0.001)	1	0.500** (*p* < 0.001)	0.480** (*p* < 0.001)
Knowledge of symptoms	0.240 (*p* = 0.064)	0.390** (*p* = 0.002)	0.510** (*p* < 0.001)	0.500** (*p* < 0.001)	1	0.460** (*p* < 0.001)
Knowledge of prevention	0.200 (*p* = 0.088)	0.440** (*p* < 0.001)	0.500** (*p* < 0.001)	0.480** (*p* < 0.001)	0.460** (*p* < 0.001)	1

**Notes:**

* Significant at the 0.05 level (2-tailed).

** Significant at the 0.01 level (2-tailed).

According to the results of this study, less than one third of the respondents selected periodic check-ins (26.2%) and laparoscopic surgery (25.5%) as treatment options for acute cholecystitis. This represents a decent amount of knowledge of both preventative care and minimally invasive surgical options. Medications were chosen by 17.4% and extracorporeal shock wave lithotripsy was selected by 14.8% of participants, which demonstrates a moderate knowledge of non-surgical options. Only a small percentage promoted open surgery (8.1%), herbal medicine (7.3%), or vague lifestyle responses like “eat fiber” or “drink plenty of water”, each accounting for less than 1%.

The results regarding medical consultation for acute cholecystitis revealed that the primary source of information was a general practitioner (34.8%). The findings indicate that 26.23% have periodic checkups, 25.45% have had laparoscopic surgery, 17.40% have used medications, and 14.81% have used lithotripsy. Overall, 84% of participants undergoing treatment or preventive treatment for acute cholecystitis.

## Discussion

The relationship between BMI, lifestyle behaviors (diet, physical activity and smoking), and knowledge of acute cholecystitis represents a vital research focus, particularly considering the increasing prevalence of this condition and its associated health effects. This cross-sectional study aimed to explore how these variables interact among adults residing in Saudi Arabia, with the goal of identifying knowledge gaps and informing targeted health education initiatives that can enhance preventive strategies and management approaches for at-risk individuals. Although the results show that participants demonstrated moderate awareness levels, significant associations were found between lifestyle behaviors, BMI, and knowledge of the condition. Increased BMI and unhealthy lifestyle behaviors were positively associated with knowledge, while greater physical activity and healthy dietary habits were positively associated with awareness of symptoms and risk factors. We believe that the findings indicate the need to combine tailored health education with lifestyle modification interventions to ease the burden of gallstone-related diseases and to promote preventive measures in Saudi Arabia.

A critical aspect of public awareness involves the ability to differentiate symptoms of biliary colic from other conditions like peptic ulcers, which require different management (Vakil, 2024). While this study focused on general knowledge, the clinical overlap between these conditions reinforces the need for accurate symptom recognition to ensure timely surgical consultation and avoid complications like pancreatitis (Kumar et al., 2025; Rodríguez-Antonio et al., 2020).

Analysis of knowledge constructs concerning acute cholecystitis indicated a moderate understanding across all domains among the participants. Specifically, awareness of risk factors and symptoms was notably higher than knowledge about prevention and dietary influences. This finding aligns with previous studies (Alghamdi et al., 2018; Mirghani et al., 2022) that have identified similar gaps in knowledge regarding preventive measures for various health conditions, such as diabetes mellitus. Patients often lack critical understanding of the impacts of dietary and lifestyle modifications, and in heart disease prevention awareness of lifestyle risk factors remains inadequate (Coughlin et al., 2020; Dai et al., 2025). While the participants generally recognized the factors contributing to the onset of acute cholecystitis and its symptoms, significant gaps remained in their understanding of preventive measures and the dietary elements that can affect their risk.

The observed variability in individual responses underscores differences in awareness of acute cholecystitis. While some participants demonstrated a thorough understanding of the condition, others exhibited limited knowledge, emphasizing the need for customized health education programs that can address these specific gaps similar to findings by Singh et al. (2023) regarding lifestyle-related disease prevention, which emphasized that health education initiatives tailored to individual knowledge levels can significantly enhance understanding and preventive behaviors (Dupont et al., 2022; Singh et al., 2023). By concentrating on dietary and preventive aspects of the disease, such initiatives can empower individuals to take proactive steps to reduce their risk of developing acute cholecystitis, ultimately enhancing their overall health outcomes.

The participants’ moderate level of understanding of the causes, symptoms, and risk factors of acute cholecystitis highlights an existing general awareness within the population, although improvements are necessary to achieve a more comprehensive understanding of potential complications and preventive lifestyle measures. A significant number of participants exhibited a high level of knowledge, demonstrating that some individuals have a well-rounded understanding of acute cholecystitis, potentially influenced by prior experiences, education, or personal interest in health topics. However, the presence of a smaller group with limited knowledge highlights an urgent need for targeted educational efforts to ensure that essential information about the condition is accessible to all.

The study findings showed that the participants’ level of education had an impact on their knowledge about acute cholecystitis, as individuals with a higher level of education were more aware of its symptoms, risk factors and measures to prevent it. This corresponds with previous research, which suggests that education promotes health literacy and leads to the uptake of behaviors designed to prevent disease. For comparison, the study by Almetrek et al. (2022) was conducted among 1,710 members of the general population in Saudi Arabia, and the study by Mirghani et al. (2022) included 1,540 participants from the Saudi general population. These findings provide useful context for interpreting the patterns observed in our study cohort (Almetrek et al., 2022; Mirghani et al., 2022). These findings emphasize the need to adapt health education programs to groups with lower educational attainment, utilizing simplified visual aids to bridge health literacy gaps.

Improving awareness may lead to earlier diagnosis, potentially reducing the high rate of emergency cholecystectomies currently seen in Saudi Arabian hospitals (Coughlin et al., 2020).

The findings of this study show significant associations between BMI, lifestyle factors and knowledge about acute cholecystitis. BMI was positively correlated with higher lifestyle scores and knowledge about dietary risks of the disease and its risk factors—those with a higher BMI were more likely to adopt a healthy lifestyle while being more informed of the possible dietary risks related to this disease. Awareness of dietary risk was also associated with awareness of risk factors, symptoms and prevention. Even though these R^2^ values are modest (<25–30%), the relationships are still useful in that they underscore relevant associations between modifiable factors and awareness levels. These findings point to the possible utility of targeted health education and lifestyle interventions to enhance knowledge, although other demographic, behavioral or psychosocial determinants are at play. Since correlation does not prove causation, future studies should explore the behavioral or psychosocial determinants that may influence these relationships. Furthermore, factors not measured here, such as family history of gallbladder disease or income level, may offer a more robust explanation for knowledge variances.

In the extant literature, Elsafi et al. (2024) conducted a study involving 300 healthcare students in Saudi Arabia and found that students with higher BMI levels demonstrated better understanding of obesity-related health issues, likely due to increased academic exposure to health education. Similarly, Gutt, Schläfer & Lammert (2020) reviewed treatment options for gallstone disease in Germany, noting that practitioners with higher BMI reported greater health awareness, potentially reflecting both personal experience and professional training. Ikeda, Ohta & Sano (2022) explored risk factors for delayed diagnosis of acute cholecystitis among older patients in Japan, concluding that individuals with higher BMI showed greater health awareness, which contributed to more prompt healthcare-seeking behaviors. These findings suggest that the positive association between BMI and health knowledge may be mediated by factors such as higher education, greater socioeconomic resources, and increased exposure to health-related information, which warrant further investigation in future studies.

Lifestyle scores were positively correlated with all four knowledge domains, emphasizing the role of lifestyle behaviors in shaping one’s understanding of acute cholecystitis. Individuals who adopt healthier lifestyles may be more inclined to seek and retain information about the condition, which could lead to a more nuanced understanding. This emphasizes the potential of lifestyle interventions to enhance knowledge and encourage preventive behaviors, a finding echoed in research by Dupont et al. (2022), which illustrates that lifestyle modifications are associated to improved health literacy (Maheta et al., 2024). Furthermore, diet control and physical activity play crucial roles in the management of gallstone disease. A balanced diet low in saturated fats and high in fiber, along with regular physical activity, can help reduce the risk of gallstone formation and manage existing conditions effectively (Ahmad et al., 2021; Liu et al., 2025). Studies indicate that consistent physical activity can reduce the risk of symptomatic gallstone disease by up to 30% to 40% (Sun et al., 2022). Educating individuals about these dietary modifications and encouraging active lifestyles can significantly contribute to the prevention and management of acute cholecystitis (Ahmad et al., 2021).

Significant correlations among the individual knowledge domains indicate that understanding in one area is linked to awareness in others. This interconnectedness implies that knowledge about acute cholecystitis is not isolated but rather holistic, with a broader understanding facilitating greater awareness across all domains. This insight supports the development of comprehensive educational strategies that address multiple facets of the disease, thereby maximizing knowledge acquisition and promoting effective prevention measures. Studies have shown that integrated educational approaches can significantly enhance knowledge retention and application (Mencarini et al., 2024; Rindler, Gries & Freidl, 2023). Mencarini et al. (2024) highlighted the importance of integrated educational approaches; they analyzed treatment strategies for acute cholecystitis with a sample of 200 healthcare providers across various clinical settings in Italy. Their findings indicated that comprehensive training significantly improved knowledge retention and practical application in clinical settings (Mencarini et al., 2024). Rindler, Gries & Freidl (2023) examined associations between overweight and mental health among 1,500 European adults aged 50+, concluding that integrated educational approaches contributed to improved awareness and understanding of both physical and mental health dimensions.

The analysis of treatment perceptions for acute cholecystitis indicates a generally informed population, revealing significant awareness of both preventive measures and surgical options. The preference for periodic check-ups and laparoscopic surgery reflects an understanding of the importance of regular monitoring and minimally invasive treatments, which are crucial for patient safety and recovery. However, the lower interest in non-surgical alternatives, such as medications and extracorporeal shock wave lithotripsy, reveals a potential gap in knowledge regarding other treatment options that could be viable prior to pursuing more invasive procedures (Singh et al., 2023).

Examining the types of healthcare providers consulted, the data show that many participants sought care from general practitioners and general surgeons, which aligns with standard practices for managing abdominal conditions. This indicates a sound understanding of the healthcare system and the appropriate specialists for acute cholecystitis management. Moreover, 6% of participants reported not consulting any physician, which raises important questions about healthcare access and self-management beliefs. This could reflect barriers to accessing healthcare, such as economic limitations or a lack of awareness of available medical resources. Additionally, it may signify an underestimation of the seriousness of the condition and a reliance on self-treatment strategies, which can lead to adverse health outcomes. Addressing these issues through targeted educational campaigns could empower individuals to seek appropriate medical advice and enhance overall management of acute cholecystitis and similar health conditions, as supported by research indicating that tailored health interventions can significantly improve healthcare-seeking behaviors (Soliman, Abdelhy & Abd El-Mageed, 2017).

## Strengths and limitations

This study provides valuable insights by examining the relationship between BMI, lifestyle behaviors, and knowledge of acute cholecystitis among adults, a topic with limited research within the local context. The cross-sectional design limits the ability to determine causal relationships between variables, including weight, height, and behaviors, are self-reported, rely on self-reported data which may introduce recall and social desirability biases. The study sample was obtained using convenience sampling through social media and digital networks, resulting in a predominantly young and female group of digitally active participants, with no representation of older adults. Consequently, the findings may not reflect the broader adult population of the region, limiting external validity and generalizability. Additionally, because the study was conducted in a specific geographic area, its generalizability to other populations may be limited, and certain confounding factors, such as dietary habits or genetic predispositions, were not thoroughly examined, which could influence the observed associations. Another limitation is that the majority of the sample were female, which may introduce gender-related bias, which could limit the generalizability of the findings. Moreover, the analyses were conducted using bivariate correlations only and did not adjust for potential confounders such as education level, socioeconomic status, health interest or healthcare access, which may explain differences in knowledge levels to a greater extent. Therefore, the findings are to be considered exploratory and hypothesis-generating to pave way for further longitudinal and analytically controlled studies.

## Conclusion

This study found that adults in Abha generally possesed a moderate level of knowledge of acute cholecystitis, with good awareness of symptoms and risk factors but limited understanding of dietary influences and preventive practices. Positive correlations were observed between BMI, lifestyle behaviors, and knowledge domains, indicate that healthier habits are associated with greater awareness. The significant interrelationship among the knowledge constructs highlight the integrated nature of health literacy. While most participants favored preventive check-ups and laparoscopic surgery, gaps persisted in awareness of alternative treatments and healthcare-seeking behaviors.

### Recommendations

Hence, causal inferences cannot be made; however, the observed knowledge gaps may assist in identifying topics of focus for future health education and research programs with the aim to improve public knowledge regarding disease symptoms, dietary risk factors and preventive behaviors. Further studies should use more representative sampling strategies, ensure balance in demographic representation, and employ either longitudinal or multivariable designs to investigate temporal and independent relationships among risk factors. Including other potentially relevant risk factors for acute cholecystitis such as socioeconomic characteristics, access to health care, genetic susceptibility and objective measures of adiposity not based on BMI may impart further insight into awareness of selected determinants and incorporate objective clinical measurements to improve data validity and reduce error measurement.

## Supplemental Information

10.7717/peerj.21172/supp-1 Supplemental Information 1 Raw Data.

10.7717/peerj.21172/supp-2 Supplemental Information 2 STROBE checklist.

10.7717/peerj.21172/supp-3 Supplemental Information 3 Questionnaire.

## Additional Information and Declarations

### Data Availability

The following information was supplied regarding data availability:

The raw data is available in the Supplemental File.
