# Supplementary material for: The association of BMI, lifestyle behaviors, and knowledge of acute cholecystitis of adults: a cross-sectional study
Source: PeerJ. 2026 May 4;14:e21172. doi: 10.7717/peerj.21172 (PMC13151927; doi:10.7717/peerj.21172)
Supplement: Supplemental Information 3 [file peerj-14-21172-s003.docx]

**Appendices**

### English version of the questionnaire

**Title:** The Association of BMI, Lifestyle Behaviors, and Knowledge of Acute Cholecystitis of adult : a cross-sectional study

**Section one: Socio-demographic characteristics and lifestyle behavior**

1. **City:**
   . Abha
   . Other
2. **Age:**
   . 18–28
   . 29–38
   . 40–50
   . 51–61
   . 62–72
   . >72
3. **Gender:**
   . Male
   . Female
4. Marital Status:
   . Married
   . Single
   . Divorced
   . Widowed
5. Educational Level:
   . No formal education
   . Primary education
   . Middle education
   . Secondary education
   . Higher education
6. Occupation:
   . Employee
   . Non-employee
   . Retired
   . Student
7. How often do you eat fried or fatty foods?
   . Daily
   . Several times a week
   . A few times a month
   . Rarely or never
8. How often do you engage in physical activity (≥150 minutes/week)?
   . Regularly
   . Occasionally
   . Rarely or never
9. Have you ever tried to lose weight for health reasons?
   . Yes
   . No
10. Height (meters): _______
11. Weight (kg): _______
12. Are you currently trying to manage or reduce your weight?
    . Yes
    . No
    . Not sure
13. Have you undergone rapid weight loss in the past year (due to extreme dieting, crash diets, or weight-loss surgery)?
    . Yes
    . No
14. Do you often skip meals or follow irregular eating patterns (e.g., long fasting, missing breakfast)?
    . Yes, frequently
    . Sometimes
    . Rarely
    . Never
15. Do you frequently consume high amounts of refined carbohydrates (such as white bread, sugary drinks, sweets, or processed snacks)?
    . Yes, very often
    . Sometimes
    . Rarely
    . Never
16. Do you currently smoke?
    . Yes

. Yes

1. Do you eat enough fruits, vegetables, and whole grains in your diet?
   . daily
   . Sometimes
   . Rarely
   . Never

18. How many hours do you sleep each night?

- a) Less than 5 hours
- b) 5–6 hours
- c) 7–8 hours
- d) More than 8 hours

19. How would you rate your stress level during the past month?

- a) Very low
- b) Low
- c) Moderate
- d) High
- e) Very high

20. How often do you consume caffeinated beverages (such as coffee, energy drinks, or tea)?

- a) Daily
- b) Several times a week
- c) Several times a month
- d) Rarely or never

21. How many meals do you typically eat per day?

- a) One meal
- b) Two meals
- c) Three meals
- d) More than three meals

### Second section: assessing the knowledge regarding dietary ingredients and habits, symptoms, and risk. Factors, prevention, and treatment of acute cholecystitis

**Part 2**: **Information about dietary ingredients and drinks that may predispose to acute cholecystitis.**

**22. Do you think consuming Fish may predispose to acute cholecystitis?**

- Yes
- No
- Don’t know

**23. Do you think consuming Beef may predispose to acute cholecystitis?**

- Yes
- No
- Don’t know

**24. Do you think consuming Coffee may predispose to acute cholecystitis?**

- Yes
- No
- Don’t know

**25. Do you think consuming Nuts may predispose to acute cholecystitis?**

- Yes
- No
- Don’t know

**26. Do you think consuming Chocolate may predispose to acute cholecystitis?**

- Yes
- No
- Don’t know

**27. Do you think smoking may predispose to acute cholecystitis?**

- Yes
- No
- Don’t know

**Part 3: Knowledge about risk factors of acute cholecystitis.**

**28. Do you think the History of AC in direct relatives increases the risk of acute cholecystitis**?

- yes
- No
- Don’t know

**29. Do you think age increases the risk of acute cholecystitis?**

- Yes
- No
- Don’t know

**30. Do you think obesity increases the risk of acute cholecystitis?**

- yes
- No
- Don’t know

**31. Do you think high cholesterol increases the risk of acute cholecystitis?**

- Yes
- No
- Don’t know

**32. Do you think high triglycerides increase the risk of acute cholecystitis?**

- Yes
- No
- Don’t know

**33. Do you think diabetes mellitus increases the risk of acute cholecystitis?**

- Yes
- No
- Don’t know

**34. Do you think high-density lipoprotein increases the risk of acute cholecystitis?**

- Yes
- No
- Don’t know

**Part 4**: **Knowledge about symptoms of acute cholecystitis**

**35. Do you think heartburn is a symptom of Acute cholecystitis?**

- Yes
- NO
- Don’t know

**36. Do you think nausea is a symptom of Acute cholecystitis?**

- Yes
- NO
- Don’t know

**37. Do you think vomiting is a symptom of Acute cholecystitis?**

- Yes
- NO
- Don’t know

**38. Do you think bloating is a symptom of Acute cholecystitis?**

- Yes
- NO
- Don’t know

**39. Do you think heaviness is a symptom of Acute cholecystitis?**

- Yes
- NO

**40. Do you think Right hypochondrium pain is a symptom of Acute cholecystitis?**

- Yes
- NO
- Don’t know

**41. Do you think intolerance to fatty meals is a symptom of Acute cholecystitis?**

- Yes
- NO
- Don’t know

**Part 5 : Knowledge about the prevention of acute** **cholecystitis.**

**42. Is acute cholecystitis prevention** **expensive**?

- Yes
- No
- Don’t know

**43. Restricting calcium intake is good for Acute cholecystitis?**

- Yes
- No
- Don’t know
- Don’t know

**44. Could dieting prevent Acute cholecystitis?**

- Yes
- No
- Don’t know

**45. Did you receive any information about Acute cholecystitis prevention?**

- Yes
- No
- Don’t know

**46. yes, what's the source of information?**

- Physicians
- Internet
- Brochures
- Tv

**47. Did this information help you?**

- Yes
- No

**Part 6: Opinions about different lines of treatment for acute cholecystitis**

**48. Which treatment option do you believe is the most effective for managing acute cholecystitis? *(Choose one option)***

- A. Laparoscopic surgery
- B. Open surgery
- C. Medications
- D. Herbs
- E. Periodic check-up
- F. Extracorporeal shock wave lithotripsy

**49. Which type of doctor did you consult about your condition or treatment?**

- A. General practitioner
- B. General surgeon
- C. Cholecystitis specialist
- D. None

**Appendices**

### Arabic version of the questionnaire

# العنوان: العلاقة بين السمنة، السلوكيات الحياتية، ومعرفة التهاب المرارة الحاد لدى البالغين

**في مدينة أبها ، منطقة عسير ، المملكة العربية السعودية: دراسة مقطعية**.

**الهدف من هذا البحث هو العلاقة**

**مقطعية مشاركتك في هذا البحث تطوعية يمكنك اختيار عدم المشاركة إن أردت. إذا قررت المشاركة في هذا البحث، يمكنك الانسحاب في أي وقت إن رغبت تتضمن المشاركة في هذا البحث ملء استبيان عبر الانترنت سيستغرق حوالي10-15 دقيقة. ستكون إجاباتك سرية ولن يتم جمع أي معلومات تعريفية**

# القسم الأول: الخصائص الاجتماعية الديموغرافية والسلوكيات الحياتية

1 المدينة:
. أبها
. أخرى

- 2 العمر:
  . أقل من 18
  . 18–28
  . 29–38
  . 40–50
  . 51–61
  . 62–72
  . أكثر من 72

3 الجنس:
. ذكر
. أنثى

4 الحالة الاجتماعية:
. متزوج
. أعزب
. مطلق
. أرمل

5 المستوى التعليمي:
. بدون تعليم رسمي
. التعليم الابتدائي
. التعليم المتوسط
. التعليم الثانوي
. التعليم العالي

6 المهنة:
. موظف
. غير موظف
. متقاعد
. طالب

7 كم مرة تتناول الأطعمة المقلية أو الدهنية؟
. يوميًا
. عدة مرات في الأسبوع
. عدة مرات في الشهر
. نادرًا أو أبدًا

8 كم مرة تمارس نشاطًا بدنيًا (≥150 دقيقة/أسبوع)؟
. بانتظام
. أحيانًا
. نادرًا أو أبدًا

9 هل سبق وحاولت إنقاص وزنك لأسباب صحية؟
. نعم
. لا

10 الطول (بالمتر): _______

11 الوزن (كجم): _______

12 هل تحاول حاليًا إدارة أو تقليل وزنك؟
. نعم
. لا
. غير متأكد

13 هل فقدت وزنًا سريعًا خلال السنة الماضية (بسبب حميات قاسية أو عمليات جراحية لإنقاص الوزن)؟
. نعم
. لا

14 هل غالبًا ما تتخطى وجبات الطعام أو تتبع أنماط أكل غير منتظمة؟
. نعم، بشكل متكرر
. أحيانًا
. نادرًا
. أبدًا

15 هل تستهلك كميات كبيرة من الكربوهيدرات المكررة؟
. نعم، كثيرًا جدًا
. أحيانًا
. نادرًا
. أبدًا

16 هل تدخن حاليًا؟
. نعم
. لا

17 هل تأكل ما يكفي من الفواكه والخضروات والحبوب الكاملة؟
. يوميًا
. أحيانًا
. نادرًا
. أبدًا

**18. How many hours do you sleep each night?**
• Less than 5 hours
• 5–6 hours
• 7–8 hours
• More than 8 hours

**19. How would you rate your stress level during the past month?**
• Very high
• High
• Moderate
• Low
• Very low

**20. How often do you consume caffeinated beverages (such as coffee, energy drinks, or tea)?**
• Daily
• Several times a week
• Several times a month
• Rarely or never

**21. How many meals do you typically eat per day?**
• One meal
• Two meals
• Three meals
• More than three meals

# القسم الثاني: تقييم المعرفة حول المكونات الغذائية والعادات والأعراض وعوامل الخطر والوقاية والعلاج لالتهاب المرارة الحاد

## الجزء 2: المعلومات حول المكونات الغذائية والمشروبات

18 هل تعتقد أن تناول السمك قد يُعرّض للإصابة بالتهاب المرارة الحاد؟
• نعم
• لا
• لا أعرف

19 هل تعتقد أن تناول اللحم البقري قد يُعرّض للإصابة بالتهاب المرارة الحاد؟
• نعم
• لا
• لا أعرف

20 هل تعتقد أن تناول القهوة قد يُعرّض للإصابة بالتهاب المرارة الحاد؟
• نعم
• لا
• لا أعرف

21 هل تعتقد أن تناول المكسرات قد يُعرّض للإصابة بالتهاب المرارة الحاد؟
• نعم
• لا
• لا أعرف

22 هل تعتقد أن تناول الشوكولاتة قد يُعرّض للإصابة بالتهاب المرارة الحاد؟
• نعم
• لا
• لا أعرف

23 هل تعتقد أن التدخين قد يُعرّض للإصابة بالتهاب المرارة الحاد؟
• نعم
• لا
• لا أعرف

## الجزء 3: المعرفة بعوامل خطر التهاب المرارة الحاد

24 هل تعتقد أن وجود تاريخ عائلي مباشر للإصابة يزيد من الخطر؟
• نعم
• لا
• لا أعرف

25 هل تعتقد أن العمر يزيد من الخطر؟
• نعم
• لا
• لا أعرف

26 هل تعتقد أن السمنة تزيد من الخطر؟
• نعم
• لا
• لا أعرف

27 هل تعتقد أن ارتفاع الكوليسترول يزيد من الخطر؟
• نعم
• لا
• لا أعرف

28 هل تعتقد أن ارتفاع الدهون الثلاثية يزيد من الخطر؟
• نعم
• لا
• لا أعرف

29 هل تعتقد أن مرض السكري يزيد من الخطر؟
• نعم
• لا
• لا أعرف

30 هل تعتقد أن ارتفاع HDL يزيد من الخطر؟
• نعم
• لا
• لا أعرف

## الجزء 4: المعرفة بأعراض التهاب المرارة الحاد

31 هل تعتقد أن حرقة المعدة من الأعراض؟
• نعم
• لا
• لا أعرف

32 هل تعتقد أن الغثيان من الأعراض؟
• نعم
• لا
• لا أعرف

33 هل تعتقد أن القيء من الأعراض؟
• نعم
• لا
• لا أعرف

34 هل تعتقد أن الانتفاخ من الأعراض؟
• نعم
• لا
• لا أعرف

35 هل تعتقد أن الشعور بالثقل من الأعراض؟
• نعم
• لا
• لا أعرف

36 هل تعتقد أن الألم في المراق اليمنى من الأعراض؟
• نعم
• لا
• لا أعرف

37 هل تعتقد أن عدم تحمل الوجبات الدهنية من الأعراض؟
• نعم
• لا
• لا أعرف

## الجزء 5: المعرفة بطرق الوقاية

38 هل الوقاية مكلفة؟
• نعم
• لا
• لا أعرف

39 هل تقييد الكالسيوم مفيد؟
• نعم
• لا
• لا أعرف

40 هل يمكن للحمية أن تمنع الالتهاب؟
• نعم
• لا
• لا أعرف

41 هل تلقيت معلومات حول الوقاية؟
• نعم
• لا
• لا أعرف

42 إذا نعم، ما المصدر؟
• الأطباء
• الإنترنت
• النشرات
• التلفزيون

43 هل ساعدتك هذه المعلومات؟
• نعم
• لا

## الجزء 6: الآراء حول الخطوط العلاجية

44 ما الخيار العلاجي الذي تعتقد أنه الأكثر فاعلية؟ (يرجى اختيار خيار واحد)
الأدوية
الأعشاب
الفحص الدوري
تفتيت الحصوات بالموجات
الجراحة المفتوحة
العمليات بالمناظير
 أخرى (يرجى التحديد)
